# Lipidomics Analysis of Outer Membrane Vesicles and Elucidation of the Inositol Phosphoceramide Biosynthetic Pathway in Bacteroides thetaiotaomicron

**DOI:** 10.1128/spectrum.00634-21

**Published:** 2022-01-26

**Authors:** Mariana G. Sartorio, Ezequiel Valguarnera, Fong-Fu Hsu, Mario F. Feldman

**Affiliations:** a Department of Molecular Microbiology, Washington University School of Medicine, St. Louis, Missouri, United States; b Division of Endocrinology, Metabolism and Lipid Research, Washington University School of Medicine, St. Louis, Missouri, United States; University of Vienna

**Keywords:** *Bacteroides*, OMV, ceramide, sphingolipids

## Abstract

Approximately one-third of the human colonic microbiome is formed by bacteria from the genus *Bacteroides*. These bacteria produce a large amount of uniformly sized outer membrane vesicles (OMVs), which are equipped with hydrolytic enzymes that play a role in the degradation of diet- and host-derived glycans. In this work, we characterize the lipid composition of membranes and OMVs from Bacteroides thetaiotaomicron VPI-5482. Liquid chromatography-mass spectrometry (LC-MS) analysis indicated that OMVs carry sphingolipids, glycerophospholipids, and serine-dipeptide lipids. Sphingolipid species represent more than 50% of the total lipid content of OMVs. The most abundant sphingolipids in OMVs are ethanolamine phosphoceramide (EPC) and inositol phosphoceramide (IPC). Bioinformatics analysis allowed the identification of the *BT1522–1526* operon putatively involved in IPC synthesis. Mutagenesis studies revealed that *BT1522–1526* is essential for the synthesis of phosphatidylinositol (PI) and IPC, confirming the role of this operon in the biosynthesis of IPC. *BT1522–1526* mutant strains lacking IPC produced OMVs that were indistinguishable from the wild-type strain, indicating that IPC sphingolipid species are not involved in OMV biogenesis. Given the known role of sphingolipids in immunomodulation, we suggest that OMVs may act as long-distance vehicles for the delivery of sphingolipids in the human gut.

**IMPORTANCE** Sphingolipids are essential membrane lipid components found in eukaryotes that are also involved in cell signaling processes. Although rare in bacteria, sphingolipids are produced by members of the phylum Bacteroidetes, human gut commensals. Here, we determined that OMVs carry sphingolipids and other lipids of known signaling function. Our results demonstrate that the *BT1522–1526* operon is required for IPC biosynthesis in B. thetaiotaomicron.

## INTRODUCTION

From birth, the mammalian gastrointestinal tract is colonized by tens of trillions of microorganisms (1, 2). The sum of all microbes within an organism, or the microbiome, has been found to be key for the development of the host immune system (3–7). Increasing evidence suggests that alterations in the mammalian colonic microbiome can influence host health and disease outcomes (8–11). Moreover, gut disease in humans has been associated with altered microbial metabolic pathways (12, 13). Approximately one-third of the human colonic microbiome is formed by bacteria from the genus *Bacteroides*, which specializes in the degradation of complex dietary polysaccharides (1, 2, 14). Bacteroides thetaiotaomicron, a common human gut commensal, has been recognized as both beneficial and detrimental to the host, according to different murine models of inflammatory disease (15–17). Components of the *Bacteroides* cell envelope, such as the capsule, proteins with hydrolytic activity, and outer membrane vesicles (OMVs), have been implicated in shaping host-symbiont interactions (17–19).

Our group has shown that a subset of proteins from *Bacteroides* spp. predicted to localize to the outer membrane (OM) are preferentially enriched in OMVs (20–22). OMVs are spherical outer membrane-derived structures that contain outer membrane and periplasmic proteins, lipids, and other molecules such as lipopolysaccharides and capsules (23–25). The proteins enriched in OMVs are mainly lipoproteins with either glycosidase or protease activity that play a role in the digestion of complex nutrients. The intrinsic properties of these proteins, such as their isoelectric points and the presence of lipoprotein export signals (LES), account for their enrichment into OMVs (21, 22). However, while *Bacteroides* OMV cargo proteins have been well described, the lipid composition of OMVs and the effect of lipids on OMV biogenesis and cargo selection remain elusive.

Although rare in bacteria, sphingolipids are produced by members of the phyla Bacteroidetes and Proteobacteria (26–29). Sphingolipids are ubiquitous and structurally diverse polar lipids, essential for eukaryotic cell membrane homeostasis (30). Different sphingolipids have been linked to a plethora of cell signaling processes, including cell death, proliferation, growth, and migration (31). These bioactive molecules have also been shown to play important roles in the development of metabolic disorders, including the ability to interfere with cholesterol absorption and insulin resistance (32–34). Furthermore, microbially derived membrane sphingolipids have been linked to reduced inflammatory disease in humans and germfree mouse models (35, 36). Species from the phylum Bacteroidetes, including the human commensals *Bacteroides* and *Porphyromonas*, synthesize sphingolipids as the main constituents of their membranes (26, 35, 37–41). Despite the knowledge that members of the human microbiota produce eukaryotic-like lipids that are associated with health and disease states, there has been a paucity of mechanistic insights into the biosynthesis of bacterial sphingolipids and their role in host-commensal interactions (26, 36, 38, 42). Mutant strains of Bacteroides fragilis and B. thetaiotaomicron unable to produce sphingolipids show lower *in vitro* stress resistance, poor colonization phenotypes, and a failure to elicit anti-inflammatory host immune responses (35, 37, 38, 40, 43).

Although these *Bacteroides*-derived lipids play a key role in host inflammatory processes, their impact on OMV biogenesis and cargo selection remains unknown. We hypothesize that specific lipids selectively partition to OMVs, where they participate in the formation and recruitment of specific protein cargo, which could impact *Bacteroides*-host interactions. To test this hypothesis, we first performed a lipidomics analysis of the total membranes (TM) and OMVs from B. thetaiotaomicron VPI-5482. Our data show that OMVs contain diverse sphingolipids, glycerophospholipids, and glycine-serine dipeptide lipids (GS). The most abundant sphingolipids are ethanolamine phosphoceramide (EPC) and inositol phosphoceramide (IPC). Genetic approaches and mass spectrometry analysis allow the identification of the IPC biosynthetic pathway. Our results indicate that IPC sphingolipids do not exert significant effects on OMV biogenesis and, indeed, OMVs might serve as IPC delivery vehicles, contributing to the regulation of host-symbiont interactions.

## RESULTS

### OMVs contain sphingolipids, glycerophospholipids, and serine dipeptide lipids.

**FIG 1:**
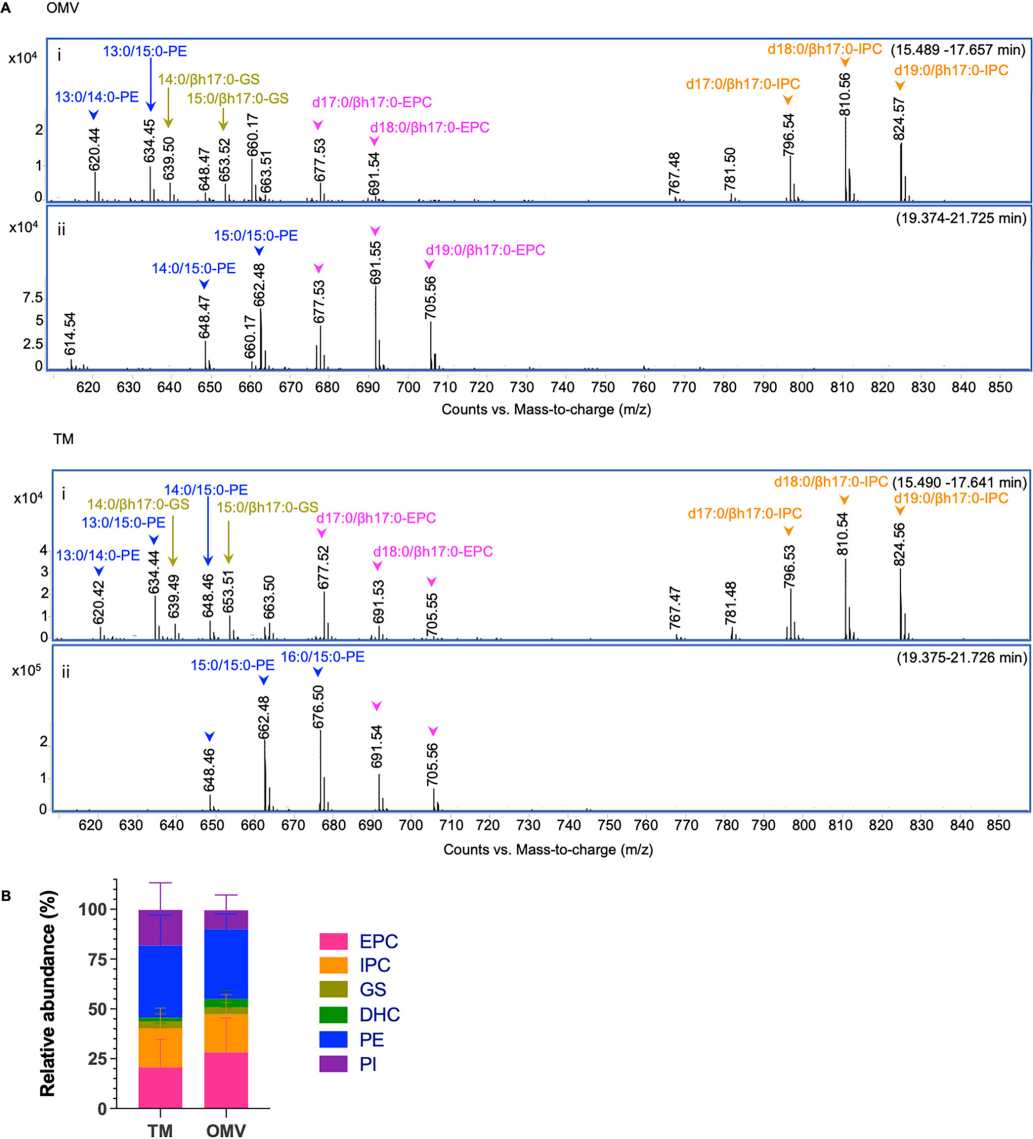
Lipid species diversity and distribution in OMVs and TM from B. thetaiotaomicron. Label-free quantification using LC-MS of lipid species from OMVs and TM of wild-type B. thetaiotaomicron. (A) ESI MS spectra showing absolute counts of the [M–H]^−^ ions of various lipids (mainly sphingonoids) from extracts of OMV (upper panels) and TM (lower panels) and the signal averaged spectra of LC fractions 15.5 to 17.6 (i) and 19.4 to 21.7 (ii) min, respectively. IPC, inositol phosphoceramide; EPC, ethanolamine phosphoceramide; PE, phosphatidylethanolamine; PI, phosphatidylinositol; DHC, dihydroceramide; GS, glycine-serine dipeptide lipids. The fatty acyl chain, e.g., “15:0/βh17:0,” indicates “C15 fatty acyl (FA) chain with no double bond” and “C17 chain with β-hydroxy side chain with no double bond,” respectively. “d17:0” represents “dihydroxy-17:0 LCB” (LCB, long-chain base). (B) Relative abundance of the most represented lipid species as a percentage of the total lipid composition for each fraction. The assigned structures (as labeled in each section in panel A) are based on tandem MS analysis of the individual ions, and the designation of the structures is according to LIPID MAPS with modifications. The graph shows the median values and standard deviation of four biological replicates.

In Bacteroidetes, the protein compositions of OMVs and the membranes from which they derive are strikingly different, with several proteins excluded from or exclusively present in OMVs (20–22, 44). We hypothesized that interaction of these proteins with lipids could be involved in the exclusion or recruitment of proteins into OMVs. In this model, it is conceivable that particular lipid species also partition between total membranes (TM) and OMVs. We employed a combination of liquid chromatography and untargeted mass spectrometry (LC-MS) to analyze the lipid contents from TM and OMVs produced by B. thetaiotaomicron VPI-5482. Mass-to-charge ratios (*m*/*z*) in negative ion mode were utilized to assign each MS peak to the most probable lipid species. When required, we employed online resources from the publicly available LIPID MAPS Lipidomics Gateway (45–47) for prediction of the lipid species based on the *m*/*z* values. We determined that B. thetaiotaomicron OMVs contain sphingolipids, glycerophospholipids, and serine dipeptide lipids (Table S1). Our analyses showed minor differences in the lipid composition of TM and OMV fractions (Fig. 1). However, it revealed interesting features of OMV lipids.

The most ubiquitous lipid class found in bacteria, glycerophospholipids, is widely represented in TM and OMV fractions from B. thetaiotaomicron by the lipid species phosphatidylethanolamine (PE) (*m*/*z* values, 620.44, 634.5, 648.5, 662.5, 676.5) and phosphatidylinositol (PI) (*m*/*z* values, 767.5, 781.5, 795.5) (Fig. 1A). A remarkable feature of lipids from Bacteroidetes is the presence of acylated amino acids (48). Acylated amino acids such as ornithine lipid and commendamide have been found in other bacterial taxonomic groups (49, 50). Acylated glycine-serine dipeptide lipids (GS) have been found and studied in the Bacteroidetes taxons *Flavobacterium* spp., Porphyromonas gingivalis, and B. thetaiotaomicron (51–55). Our data showed that GS (*m*/*z* values, 639.5, 653.5) were present in both B. thetaiotaomicron TM and OMV fractions (Fig. 1B).

The most abundant lipid species found in OMVs were sphingolipids, mainly ceramide (*m*/*z* values, 600.4, 614.5, 628.5) and ceramide-derived compounds, including ethanolamine phosphoceramide (EPC) (*m*/*z* values, 663.5, 677.5, 691.5, 705.6) and inositol phosphoceramide (IPC) (*m*/*z* values, 796.5, 810.5, 824.6) (Fig. 1A; Table S1). EPC has been found in B. fragilis, B. thetaiotaomicron, and Bacteroides ovatus (56). IPC, a sphingolipid typically found in eukaryotes, has been discovered in B. thetaiotaomicron and *B. ovatus* (35, 38, 43). However, neither of the biosynthetic pathways of these sphingolipids has been elucidated.

### Genetic basis of IPC synthesis.

**FIG 2:**
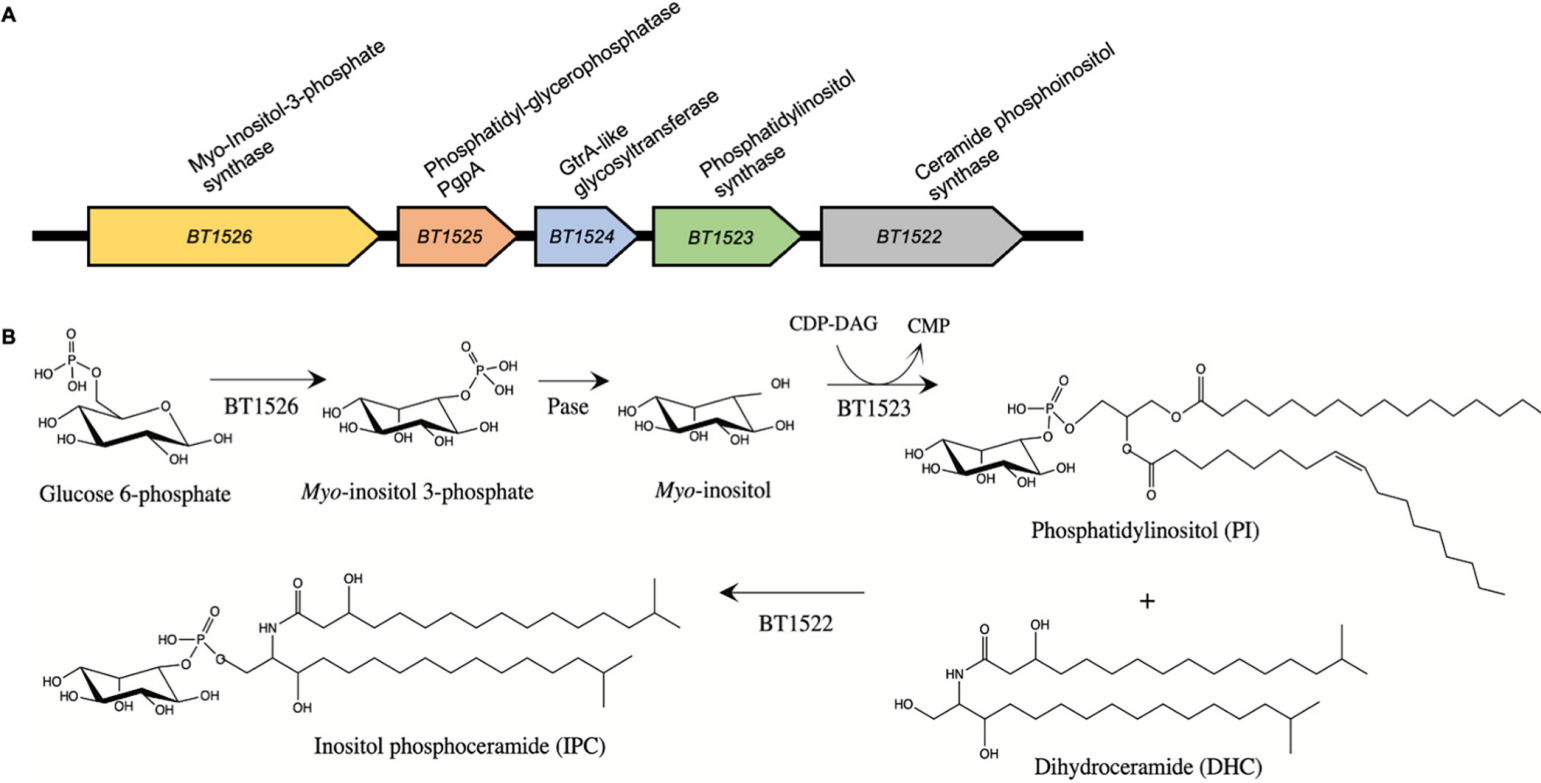
Proposed genes and pathway for IPC synthesis. (A) Schematic representation of the *BT1522*–*1526* hypothetical IPC biosynthesis operon with the predicted gene function estimated by PSI-BLAST, HHpred, and Phyre2 analysis. (B) Schematic overview of the predicted IPC biosynthetic pathway. Pase, phosphatase.

**FIG 3:**
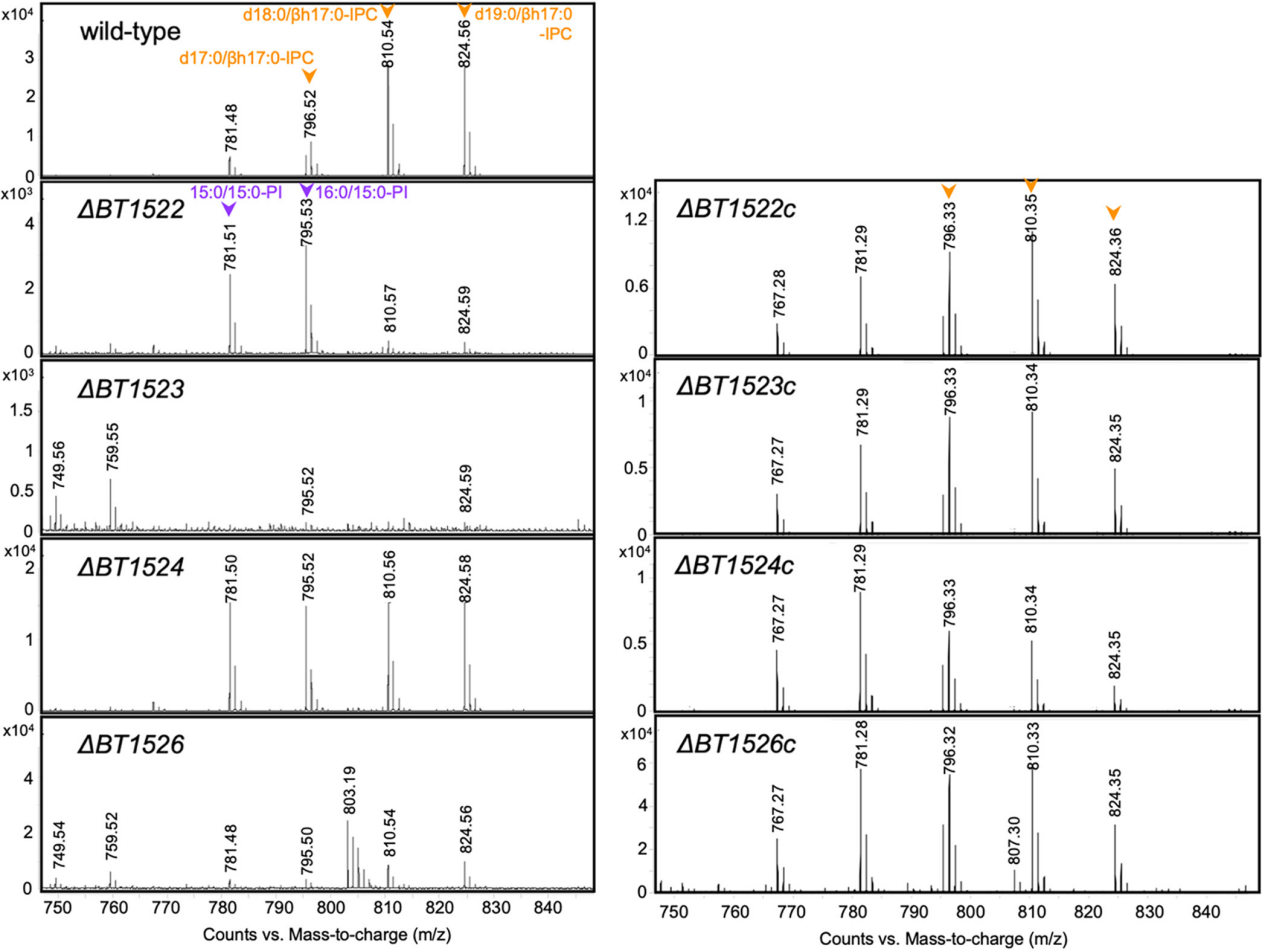
ESI MS spectra showing absolute counts of the [M–H]^−^ ions of IPC and PI molecules of TM lipids extracted from wild-type and *BT1522–1526* mutants (left panel) and their complemented strains (right panel). The orange arrows indicate IPC lipid species; the purple arrows indicate PI lipid species. The graphs are representative of three biological replicates.

**FIG 4:**
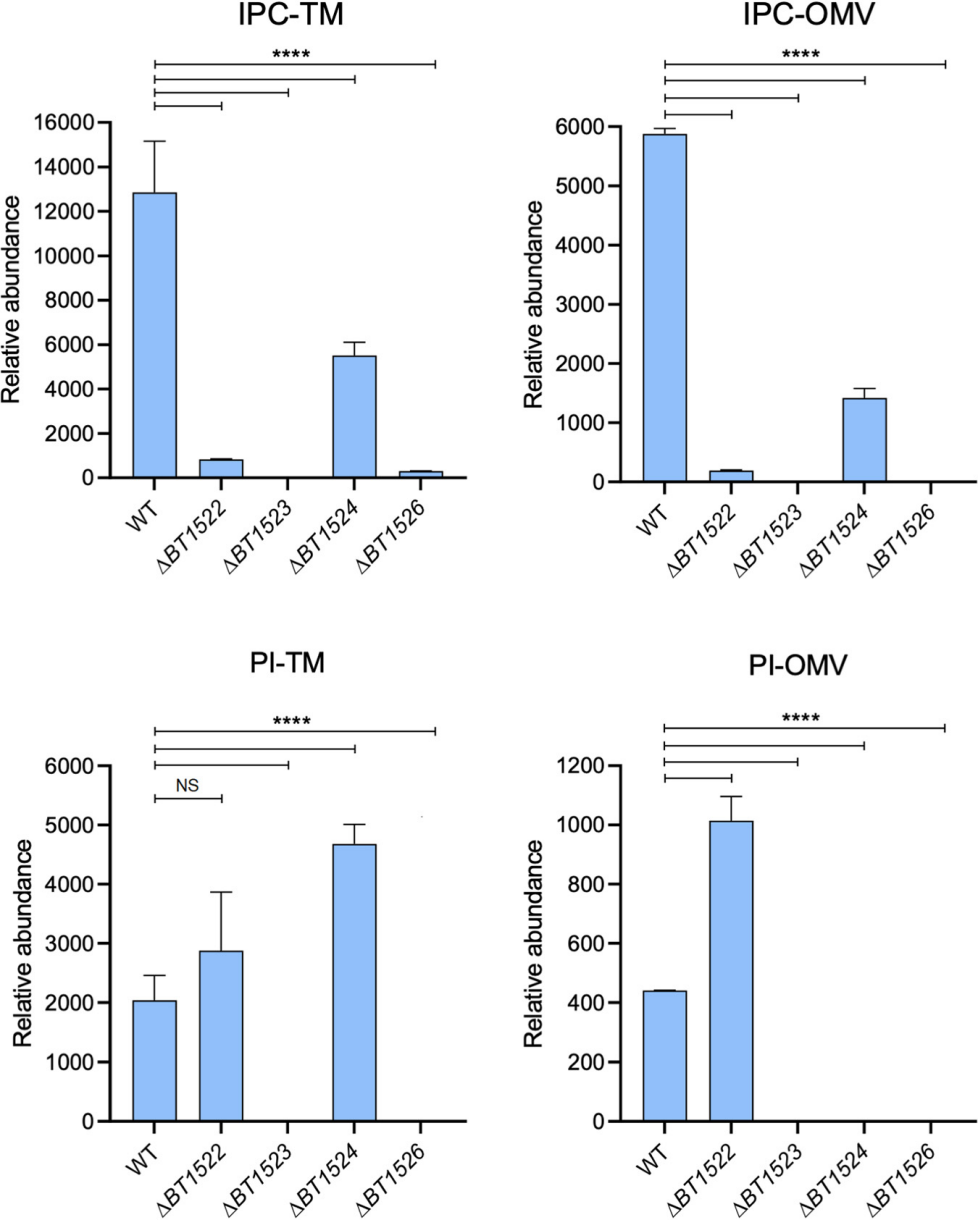
Relative abundances of IPC and PI species from TM and OMV fractions. The graphs show the median values of two biological replicates with the range for individual lipid species from each sample. Analysis of variance (ANOVA) tests were used to determine the statistical significance with an alpha value of 0.05; ****, *P* < 0.0001.

We postulated that sphingolipids may be involved in OMV biogenesis and sought to study OMVs in a mutant strain unable to produce this class of lipids. The first step in sphingolipid biosynthesis is the synthesis of 3-dehydroxysphinganine using palmitoyl-coenzyme A (CoA) and serine as the substrates via serine palmitoyl transferase (SPT). SPT orthologs are conserved in Bacteroidetes and carry the same function as in eukaryotes (35, 37, 39, 40, 43). Recent work has identified the *spt* ortholog in B. thetaiotaomicron, *BT0870*, and shown its essential function in sphingolipid biosynthesis (35). We generated a deletion strain for *spt* in B. thetaiotaomicron using a thymidine kinase (*tdk*) mutant strain as our wild-type (WT) genetic background, allowing for negative selection of transconjugants (57). Deletion of *spt*, as expected, resulted in a B. thetaiotaomicron strain unable to synthesize sphingolipids, as indicated by LC-MS (Fig. S1). The complete absence of sphingolipids caused a drastic reduction in bacterial growth and lysis, which made analysis of the contribution of OMV sphingolipids in B. thetaiotaomicron unfeasible (Fig. S1). Thus, we sought to investigate the roles of specific sphingolipids, such as EPC and IPC, in OMV formation. We performed BLAST analyses against the B. thetaiotaomicron VPI-5482 proteome using amino acid sequences from eukaryotic EPC and IPC synthases (CPES from Drosophila melanogaster and AUR1 from Saccharomyces cerevisiae). While no matches were found for EPC synthase, we found a putative ortholog in B. thetaiotaomicron, *BT1522*, predicted to encode an IPC synthase. Analysis of the genes surrounding *BT1522* suggested that *BT1522* is part of an operon composed of genes *BT1522* to *BT1526* (35). We employed PSI-BLAST, HHpred, and Phyre2 to interrogate the function of the predicted proteins encoded by the *BT1522–1526* operon (Fig. 2A) and generate a predicted IPC biosynthetic pathway (Fig. 2B) (58–60). *BT1526* is annotated as a myo-inositol-phosphate synthase, an enzyme that generates myo-inositol-3-phosphate from glucose-6-P (Fig. 2B) (61, 62). Myo-inositol 3-phosphate is the building block for most phosphoinositol-derived compounds (61, 62). *BT1525* is predicted to encode a phosphatidylglycerol-1-phosphate (PGP) phosphatase, an enzyme that generates phosphatidylglycerol using PGP as the substrate (Fig. 2A) (63). *BT1524* is predicted to encode a GtrA-like protein, an inner membrane protein that seems to be required for synthesis of lipid-linked glycans in bacteria (64–66). *BT1523* is predicted to encode a phosphatidylinositol (PI) synthase, which utilizes inositol and cytidine diphosphate (CDP)-diacylglycerol to synthesize phosphatidylinositol (Fig. 2B) (67). Finally, an IPC synthase (*BT1522*) generates IPC in eukaryotes using ceramide and phosphatidylinositol as the substrates (Fig. 2B) (68, 69). To generate clean deletion mutant strains for genes *BT1522* to *BT1526*, we employed the thymidine kinase (*tdk*) mutant strain as our wild-type genetic background. We were able to obtain mutant strains for genes *BT1522*, *BT1523*, *BT1524*, and *BT1526*. Deletion mutants for the *BT1525* gene could not be obtained after many rounds of negative selection, suggesting that *BT1525* is an essential gene in B. thetaiotaomicron. Lipid analyses by LC-MS of TM and OMVs showed no IPC content in the *BT1523* mutant and negligible IPC levels (<1% relative abundance) for the *BT1522* and *BT1526* mutants (Fig. 3, Fig. S2; Table S1) in both the TM and OMV fractions (Fig. 4). The *BT1524* mutant strain showed significantly lower IPC relative abundance than the wild-type strain in both fractions (Fig. 4). Conversely, the *BT1524* mutant displayed higher relative abundance levels of PI in the TM than the wild-type strain (Fig. 4), suggesting that *BT1524* impacts the synthesis of IPC through an unknown mechanism. PI was not detected in the *BT1523* and *BT1526* mutants in either fraction, in agreement with their predicted functions in PI biosynthesis. The *BT1522* mutant strain displayed relative abundance levels of PI indistinguishable from those of the wild-type strain (Fig. 4). In agreement with abolished IPC synthesis, membranes from the *BT1522*, *BT1523*, and *BT1526* strains displayed higher levels of ceramides than wild-type and *BT1524* mutant strains (Table S1). The mutants in the IPC displayed mild growth defects (Fig. S3). Complementation of the *BT1522–1526* mutated genes restored the IPC lipid profiling in all the individual mutants (Fig. 3, right panel), confirming that the operon is necessary and sufficient for IPC biosynthesis in B. thetaiotaomicron.

### Depletion of IPC does not affect OMV biogenesis or cargo.

**FIG 5:**
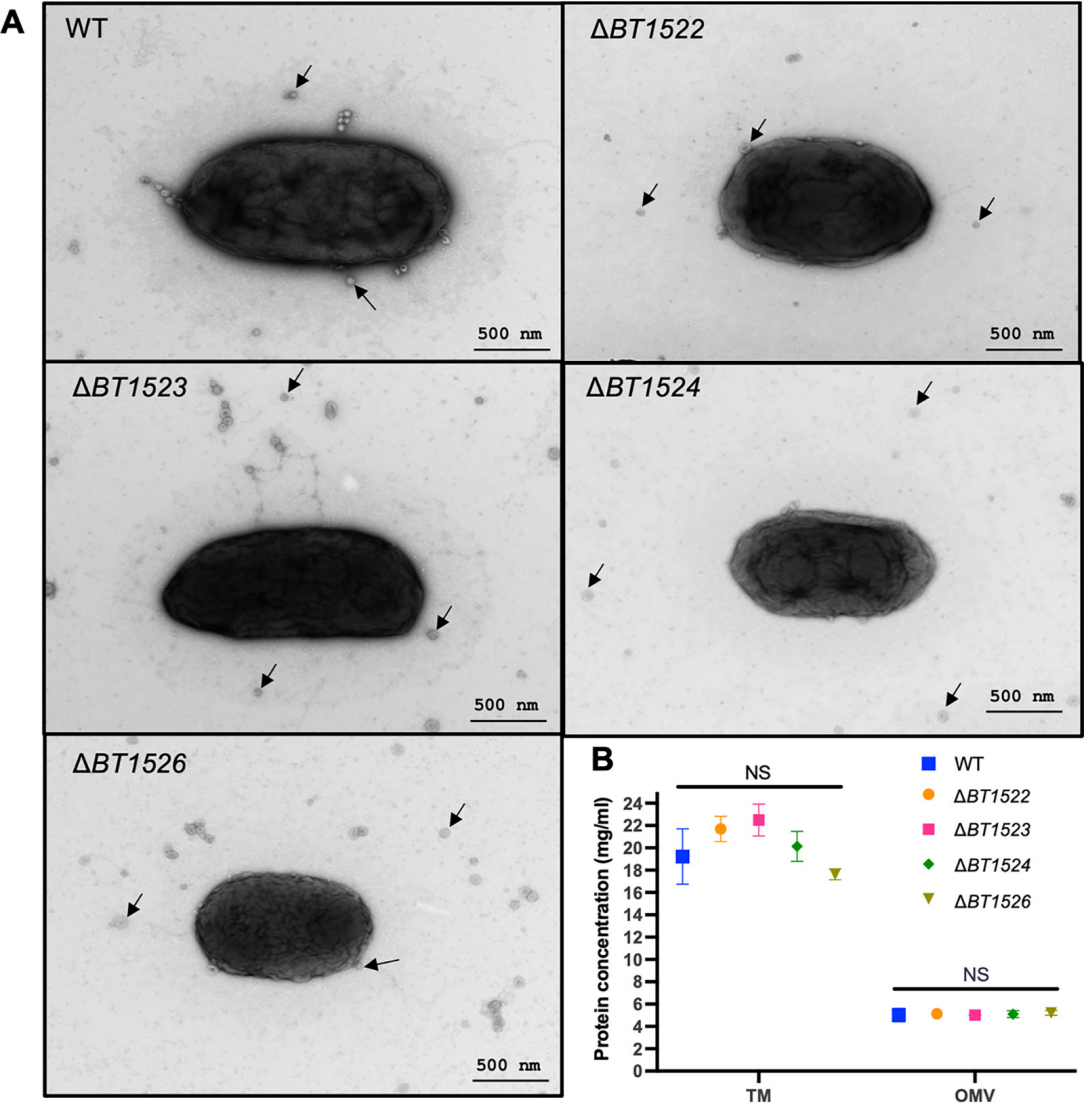
(A) Transmission electron microscopy of B. thetaiotaomicron wild-type (WT) and Δ*BT1522–1526* strains showing no significant differences in shape. The strains were grown on BHI agar plates, and bacterial lawns were swabbed from the surface of the plates and resuspended in PBS for imaging by TEM. These images were acquired from Wandy Beatty at the WUSTL Molecular Microbiology Imaging Facility. Magnification of 20,000× is shown. (B) TM and OMV fractions were prepared using early stationary-phase liquid-grown cultures of WT and Δ*BT1522–1526* strains. The TM and OMV preparations were resuspended in PBS for each strain, and the total protein concentration was determined. The graph shows the means and error bars by standard deviation (SD) of 3 biological replicates for each strain. Statistical significance was determined by *t* test (*P* value, <0.05).

**FIG 6:**
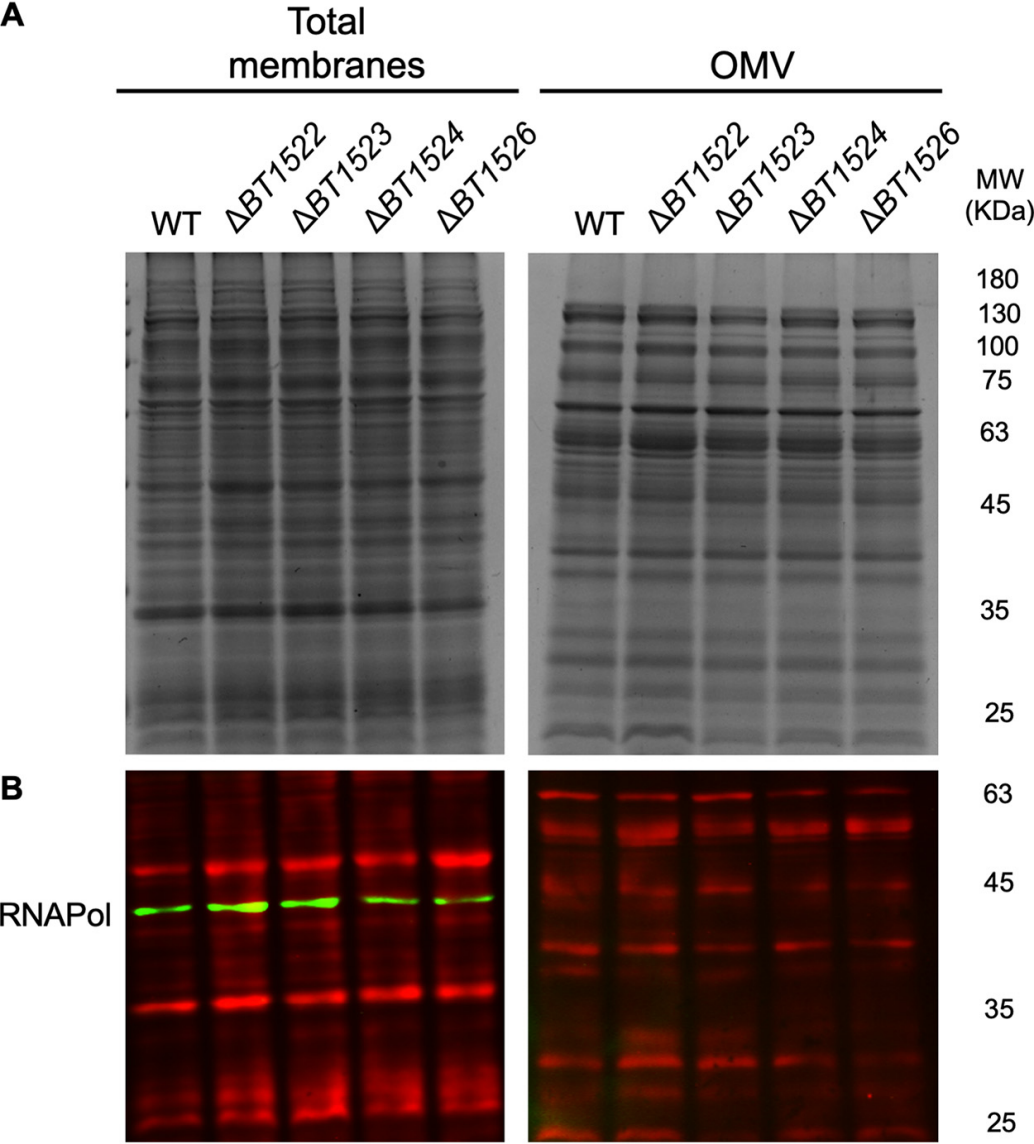
TM and OMV fractions were prepared using early stationary-phase liquid-grown cultures. The TM and OMV preparations were resuspended in PBS, and the protein concentration was determined: 10 μg protein per sample was loaded onto 12% SDS-PAGE gels and analyzed by (A) Coomassie blue staining or (B) Western blot, using anti-RNA polymerase antisera and REVERT total protein stain as the loading control. Lack of IPC does not cause cell lysis or impact the differential TM/OMV protein profile.

IPC could be required for OMV biogenesis, or alternatively it could mediate interactions with proteins, impacting the OMV cargo. We analyzed B. thetaiotaomicron cells and OMV preparations using transmission electron microscopy (TEM) and found no obvious differences in morphology between wild-type and IPC mutant strains (Fig. 5A; Fig. S4). We also found similar levels of vesiculation, vesicles sizes, and OMV protein concentration among all strains (Fig. S4 and Fig. 5B). Analysis of TM and OMV protein contents using SDS-PAGE and Coomassie blue stain showed indistinguishable protein band patterns between the wild-type and *BT1522–1526* mutant strains (Fig. 6A). Western blot assays of the TM and OMV fractions revealed that, despite their mild growth defects, the mutant strains do not lyse spontaneously, as evidenced by the lack of RNA polymerase signal in OMV preparations (Fig. 6B).

As mentioned, *Bacteroides* preferentially packs OMVs with certain lipoproteins that are mainly acidic and have hydrolytic activity, resulting in the selection of cargo components and the exclusion of other abundant OM proteins (21, 22, 44). To examine whether *BT1522–1526* mutant strains have an impact on protein cargo selection, we analyzed SusG secretion into OMVs in the different strains. SusG is a lipoprotein with amylase activity that has previously been shown to be preferentially packaged into OMVs (22). Western blot assays of TM and OMV fractions of the different mutants revealed no differences in SusG packaging into OMVs (Fig. S5). Together, these data rule out a major role for IPC in OMV formation or cargo selection.

## DISCUSSION

In this work, we determined the lipid composition of OMVs produced by B. thetaiotaomicron. LC-MS analysis demonstrated that sphingolipid species represent more than 50% of the total lipid content of OMVs. Among these, EPC and IPC were the most abundant sphingolipids. We determined that the *BT1522-1526* operon is essential for the synthesis of PI and IPC, the latter being the second most abundant sphingolipid species in OMV fractions. *BT1522–1526* mutant strains lacking IPC displayed mild growth defects, but their OMV secretion phenotypes were indistinguishable from that of the wild-type strain. Furthermore, protein cargo selection into OMVs was not affected in the different mutants analyzed, indicating that IPC is not required for OMV biogenesis.

The results from this study indicate that the protein encoded by *BT1522* is the main IPC synthase in B. thetaiotaomicron. Remarkably, the detection of very low levels of IPC in membranes from the *BT1522* mutant strain suggests partial redundancy by an unknown enzyme that could be acting as an IPC synthase in a suboptimal manner. Moreover, higher levels of dihydroceramide and dehydrated ceramide (Table S1) in the *BT1522*, *BT1523*, and *BT1526* mutants in comparison to those in the wild-type and *BT1524* strains strongly suggest that these lipid molecules are the precursors for IPC synthesis in B. thetaiotaomicron. Intriguingly, our results have also shown that in the *BT1524* mutant, the PI accumulation in TM was not reflected in the OMV fraction, as was observed in the *BT1522* mutant (Fig. 4, lower panels). Future work will help to elucidate the function of *BT1524* in IPC synthesis and whether it is linked to the selection of OMV lipid cargo in B. thetaiotaomicron.

Regarding the impact of PI/IPC on cell growth, our results suggest that while a lack of IPC partially impacts the exponential growth phase, its absence could have a role in survival after reaching the stationary phase. P. gingivalis, B. fragilis, and B. thetaiotaomicron lacking SPT activity display a reduction in growth and post-stationary-phase viability, suggesting that IPC could be mediating this phenotype in B. thetaiotaomicron (37, 39).

Regarding other lipids with potential signaling activities within the host milieu, our results showed very similar levels of GS between TM and OMVs. A recent study has shown that the gene *glsB* (*BT3459*) from B. thetaiotaomicron is required for the synthesis of GS, providing a partial genetic basis for amino lipid production in Bacteroidetes (55). Similar to sphingolipids, GS are also required for bacterial homeostasis and colonization of the murine gut (55). A specific serine-containing lipid from P. gingivalis, called lipid 654, can be cleaved by host phospholipases into lipid 430, a highly inflammatory derivative (52). Amino lipids signal inflammation through host TLR2 receptors and have been proposed as markers of atherosclerosis (52, 53, 70, 71). Nevertheless, the potential contributions of amino lipids to OMV formation and gut inflammation by B. thetaiotaomicron are still unknown. The majority of B. thetaiotaomicron cells reside within the colonic mucus layer in mice, and a small proportion localize within colonic crypts in close association to epithelial cells (72–76). Data from irritable bowel disease (IBD) patients show that a decrease in *Bacteroides* sphingolipids is negatively correlated with an increase in host ceramides, in comparison to a healthy human cohort (13, 35). Recent data from the Ley group has showed that sphingolipids from *Bacteroides* are likely to be incorporated *in vivo* into colonic epithelial cells and modify the host glucose metabolism (77). *In vitro* studies in P. gingivalis also showed the incorporation of bacterial sphingolipids into THP I human cell lines in a contact-independent manner and a decreased inflammatory response from the host cell compared to that in SPT-deficient strains (40, 78). Altogether, these results indicate that sphingolipid-enriched OMVs act as carriers of secreted anti-inflammatory signals within the colonic niche and are taken up by host colonocytes (79). This mechanism of sphingolipid delivery could bypass the distance imposed by the colonic mucus barrier between *Bacteroides* cells and colonic epithelial cells. Our data, along with the work of others, show an opportunity to use targeted sphingolipid-deficient bacterial strains to genetically dissect the contribution of the *Bacteroides* sp. cell envelope to colonic homeostasis and gut symbiosis. B. fragilis, the *Bacteroides* species most frequently isolated from anaerobic infections in humans, lacks a *BT1522–1526* orthologous region in its genome. Gain-of-function experiments incorporating genes from the *BT1522–1526* region into a different *Bacteroides* species such as B. fragilis could contribute to dissecting the role of IPC synthesis genes in a heterologous context. While sphingolipids are the main lipid components of *Bacteroides* sp. membranes, we and others have shown that lipid repertoires vary according to the species analyzed (35, 37, 38, 43, 77). We thus hypothesize that any contributions of *Bacteroides* sphingolipids to health or disease states will likely be governed by the species composition and relative abundance within each host microbiome.

## MATERIALS AND METHODS

### Bacterial strains and growth conditions.

Oligonucleotides, strains, and plasmids are described in Table S2 in the supplemental material. Escherichia coli strains were grown in lysogenic broth (LB) or on LB agar plates. *Bacteroides* strains were grown in an anaerobic chamber (Coy Laboratories) using an atmosphere of 10% H_2_, 5% CO_2_, 85% N_2_. For liquid and solid growth, brain heart infusion (BHI) or BHI agar plates supplemented with hemin and vitamin K3 were used. Antibiotics and other compounds were used as follows: ampicillin at 100 μg/mL, erythromycin at 25 μg/mL, and bromodeoxyuridine at 200 μg/mL. For growth curves, overnight cultures of B. thetaiotaomicron wild-type and mutant strains were back-diluted into 96-well plates using BHI equilibrated to an anaerobic atmosphere. A final volume of 0.2 mL per well was used, and the optical density at 600 nm (OD_600_) was adjusted to 0.1. Plates containing an anaerobic atmosphere were sealed with non-gas permeable transparent film and incubated at 37°C inside a temperature-controlled plate reader (Synergy HTX, BioTek Instruments). OD_600_ measurements were taken every 15 min.

### Construction of plasmids, mutagenesis, generation of mutants by clean deletion and complementation.

Generation of B. thetaiotaomicron deletion strains was carried out using the Δ*tdk* strategy as previously described (57). Briefly, 1,000-bp upstream and downstream fragments of *BT0870*, *BT1522*, *BT1523*, *BT1524*, *BT1525*, and *BT1526* were cloned into the pExchange-*tdk* vector using E. coli S17-1 λpir as the cloning strain. Constructs were conjugated into B. thetaiotaomicron Δ*tdk* cells using previously transformed E. coli S17-1 λpir as the donor, and strain plating and selection were performed as previously described (57).

For complementation of the mutant strains, *BT1522*, *BT1523*, *BT1524*, and *BT1526* were PCR amplified and purified. Each fragment was cloned into the pWW3867 integrative plasmid backbone using the Gibson DNA assembly method (NEB) (72). E. coli S17-1 λpir was used as the cloning strain, and each construct was conjugated into the corresponding B. thetaiotaomicron Δ*BT1522–1526* mutant strains (Table S2). Positive transformants were selected by antibiotic resistance as described previously (57).

### OMV preparations.

Crude OMVs were obtained by ultracentrifugation of the filtered spent medium from 150 mL of liquid culture as described (21). Briefly, 18- to 20-h cultures of B. thetaiotaomicron were centrifuged at 6,500 rpm at 4°C for 10 min. To remove residual cells, the supernatant was filtered using a 0.22-mm-pore membrane (Millipore). The filtrate was subjected to ultracentrifugation at 200,000 × *g* for 2 h (Optima L-100 XP ultracentrifuge; Beckman Coulter). The supernatant was discarded, and the pellets containing the OMV preparation were resuspended in phosphate-buffered saline (PBS) and normalized by OD before protein and lipid analyses. The OMV amount was estimated by measuring the protein content using a DC protein assay kit (Bio-Rad). Fractions were aliquoted and stored at −80°C until analyzed.

### Membrane preparations.

Total membrane preparations were obtained by cell lysis and ultracentrifugation as previously described (21). The total membranes from 150 mL of liquid culture were resuspended in PBS using a 2-mL glass tissue grinder with a polytetrafluoroethylene (PTFE) pestle (VWR). The protein content was quantified using a DC protein assay kit (Bio-Rad). The fractions were aliquoted and stored at −80°C until analysis.

### Total lipid extractions.

Total lipids from OMVs and TM were extracted based on the Bligh and Dyer chloroform:methanol method (80). Briefly, 2 volumes of methanol, 1 volume of chloroform, and 0.8 volumes of Milli-Q water were added to 1 volume of PBS-resuspended OMV or TM fractions in solvent-resistant glass tubes. The contents were mixed for 1 min by vortexing, and 1 volume of chloroform was added to the mixture. The contents were mixed for another minute, and the tubes were centrifuged for 5 min at 4,000 rpm. After centrifugation, the bottom phase (organic) was recovered using a glass Pasteur pipette and stored in solvent-sealed vials at −80°C until lipid analysis by LC-MS.

### LC-MS analysis of lipids from TM and OMVs.

Untargeted LC/MS analyses were conducted on an Agilent 6550 A QTOF instrument with an Agilent 1290 high-performance liquid chromatograph (HPLC) with an autosampler, operated using Agilent MassHunter software (Santa Clara, CA, USA). Separation of the total lipid extracts was achieved using a Thermo Fisher (Waltham, MA, USA) BETASIL C_18_ column (100 × 2.1 mm, 5 μm) at a flow rate of 300 μL/min at room temperature. The mobile phase contained 5 mM ammonium formate (pH 5.0) both in solvent A, acetonitrile:water (60:40, vol/vol), and solvent B, isopropanol:acetonitrile (90:10, vol/vol). A gradient elution was applied in the following manner: 68% A, 0 to 1.5 min; 68 to 55% A, 1.5 to 4 min; 55 to 48% A, 4 to 5 min; 48 to 42% A, 5 to 8 min; 42 to 34% A, 8 to 11 min; 34 to 30% A, 11 to 14 min; 30 to 25% A, 14 to 18 min; 25 to 3% A, 18 to 23 min; 3 to 0% A, 25 to 30 min; 0% A, 30 to 35 min; 68% A, 35 to 40 min. Both the positive-ion and negative-ion electrospray ionization (ESI) MS scans were acquired in the mass range of 200 to 2,000 Da at a rate of 2 scans/min. High-resolution (*R* = 100,000 at *m*/*z* 400) mass spectrometric analyses of the lipid extracts were also conducted on a Thermo LTQ Orbitrap Velos. Lipids were loop injected into the ESI ion source using a built-in syringe pump which was set to continuously deliver a flow of 20 μL/min methanol with 0.5% NH_4_OH. The scanned mass spectra were recalibrated internally with a known mass, namely, 13:0/15:0 PE at *m*/*z* 634.4453. Linear ion trap (LIT) multistage MS (MS^n^) spectra were obtained for structural identification as described previously (81–83).

### Transmission electron microscopy.

For negative staining and analysis by TEM, bacterial suspensions in PBS or OMV were allowed to absorb onto freshly glow-discharged Formvar/carbon-coated copper grids for 10 min. The grids were washed in distilled water and stained with 1% aqueous uranyl acetate (Ted Pella, Inc., Redding, CA) for 1 min. Excess liquid was gently wicked off, and the grids were allowed to air dry. The samples were viewed on a 1200EX transmission electron microscope (JEOL USA, Peabody, MA) equipped with an 8-megapixel digital camera (Advanced Microscopy Techniques, Woburn, MA).

### SDS-PAGE and Western blot analysis.

The total membrane and OMV fractions were analyzed using standard 12% Tris-glycine SDS-PAGE gels. Briefly, 10 μg of TM or OMV fractions were loaded onto SDS-PAGE gels in duplicate. One gel was stained with Coomassie blue to visualize the protein band patterns. The other gel was transferred onto a 0.45-μm nitrocellulose membrane (Bio-Rad), and Western blotting was performed using the LI-COR system. After transfer of the protein preparations from the SDS-PAGE gels, nitrocellulose membranes were incubated with REVERT total protein stain as described by the manufacturer (LI-COR) and imaged immediately at 680 nm. The membranes were blocked using Tris-buffered saline (TBS)-based 3% nonfat milk blocking solution. The primary antibodies used in this study were mouse monoclonal anti-E. coli RNA polymerase (RNApol) subunit alpha (BioLegend). The secondary antibodies used were IRDye antimouse 780 antibodies (LI-COR). Imaging was performed using an Odyssey CLx scanner (LI-COR).

### Data availability.

The resulting lipidomic data are available at the NIH Common Fund’s National Metabolomics Data Repository (NMDR) website, https://www.metabolomicsworkbench.org (84), where they have been assigned project ID PR001199. The data can be accessed directly via https://doi.org/10.21228/M8QQ5J.
