# Supplementary material for: Lipidomics Analysis of Outer Membrane Vesicles and Elucidation of the Inositol Phosphoceramide Biosynthetic Pathway in Bacteroides thetaiotaomicron
Source: Microbiol Spectr. 2022 Jan 26;10(1):e00634-21. doi: 10.1128/spectrum.00634-21 (PMC8791184; doi:10.1128/spectrum.00634-21)
Supplement: SUPPLEMENTAL FILE 1 — Supplemental material. Download SPECTRUM00634-21_Supp_1_seq10.pdf, PDF file, 3.0 MB [file spectrum00634-21_supp_1_seq10.pdf]

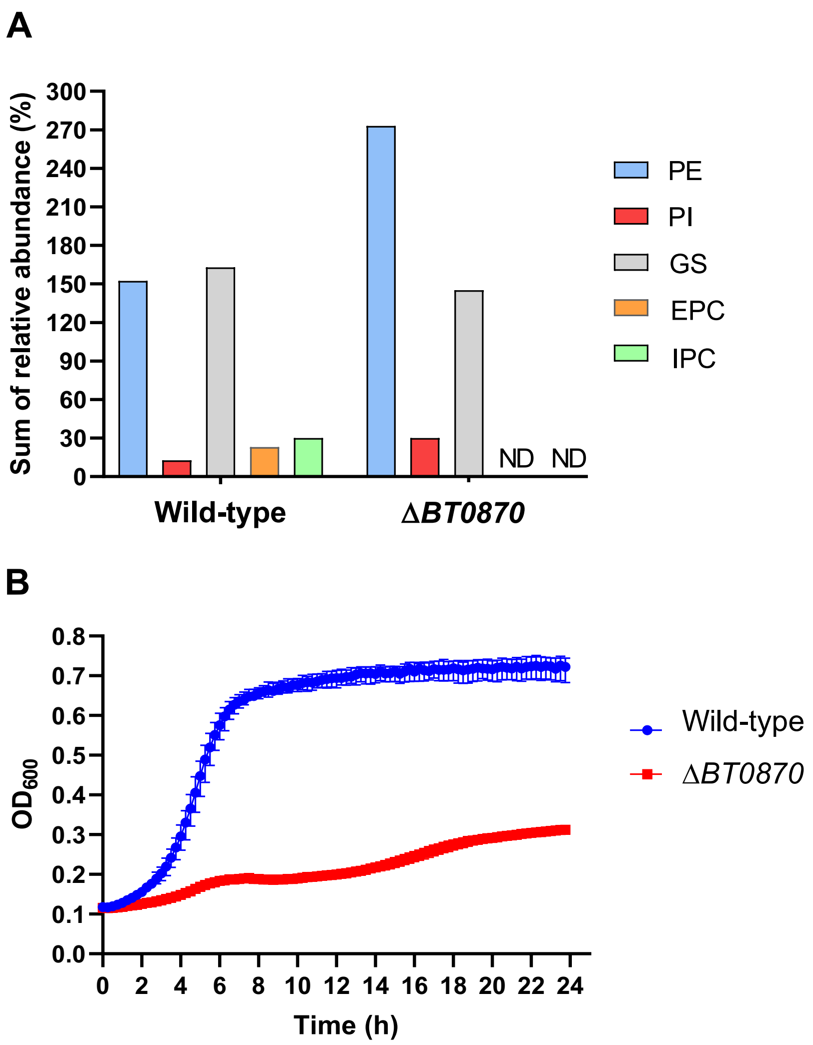

Figure S1. BT0870 is essential for sphingolipid production in *B. thetaiotaomicron*. (A) Sum of relative abundances of different lipid species determined by MS from TM fraction. Graphs show median values for individual lipid species from each sample, relativized to the most abundant lipid species (assigned 100% value); PE: phosphatydilethanolamine; PI: phosphatydilinositol; GS: glycylserine peptidoli; EPC: ethanolamine phosphoceramide; IPC: Inositol phosphoceramide; ND: not detected **(B)** Growth curve in liquid BHI media of wild-type (WT) and Δ*BT0870* strains. Cells were grown at 37°C in an anaerobic atmosphere and optical density at 600 nm was determined every 15 min.

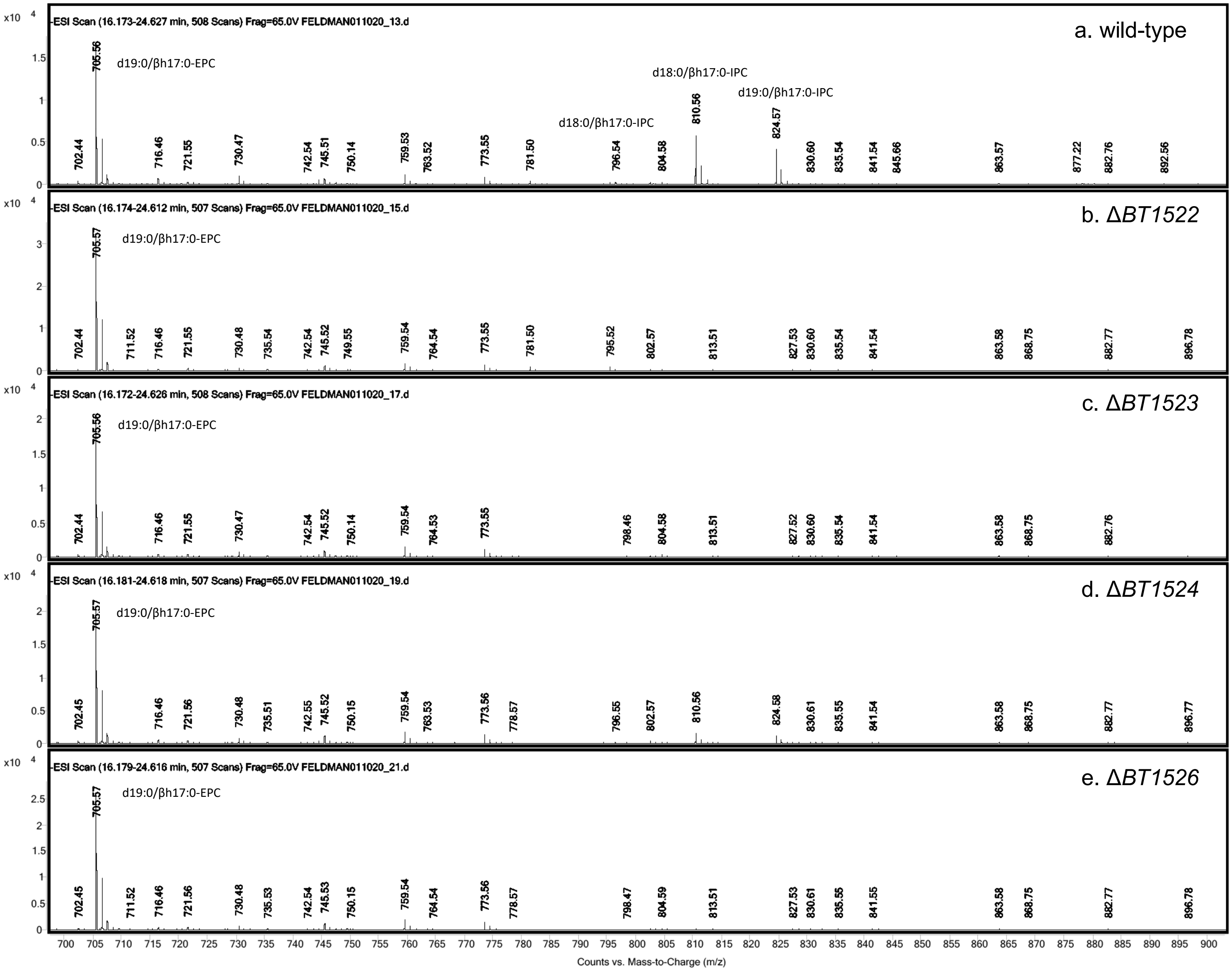

Figure S2. The *BT1522-1526* region is essential for synthesis of IPC. The MS spectra of a) WT, b) *ΔBT1522*, c) *ΔBT1523*, d) *ΔBT1524*, e) *ΔBT1526* for OMV lipids plotted from LC fraction 15.9-17.3 min showing the presence of three major IPC species (i.e., m/z 796, 810, and 824) in WT, while these species are almost absent in panels b-e. The coeluted ions of d19:0/βh17:0-EPC at m/z 705 are present in all samples (panels a-e) to illustrate EPC abundance remains stable in the different strains analyzed.

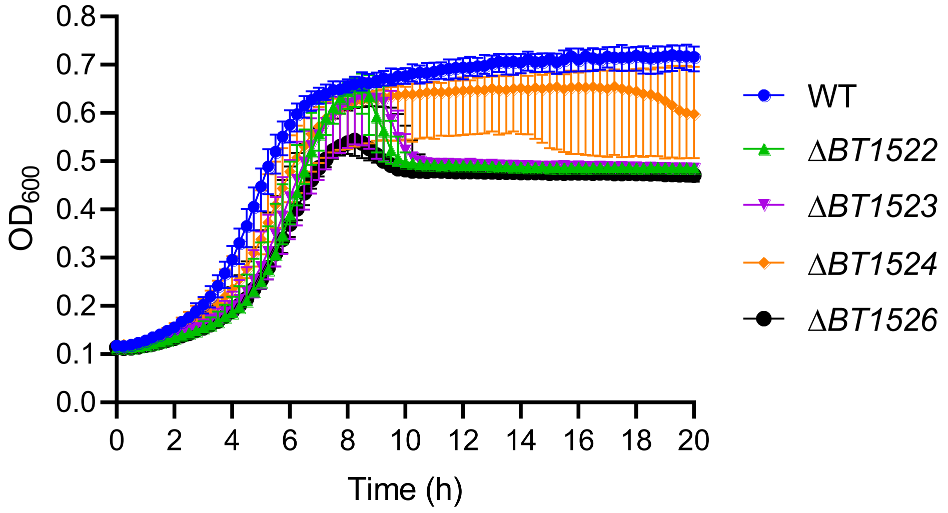

Figure S3. Lack of IPC and PI produces mild growth defects in *B. thetaiotaomicron in vitro*. Growth curve in liquid BHI media of wild-type (WT) and Δ*BT1522-BT1526* strains. Cells were grown at 37°C in an anaerobic atmosphere and optical density at 600 nm was determined every 15 min.

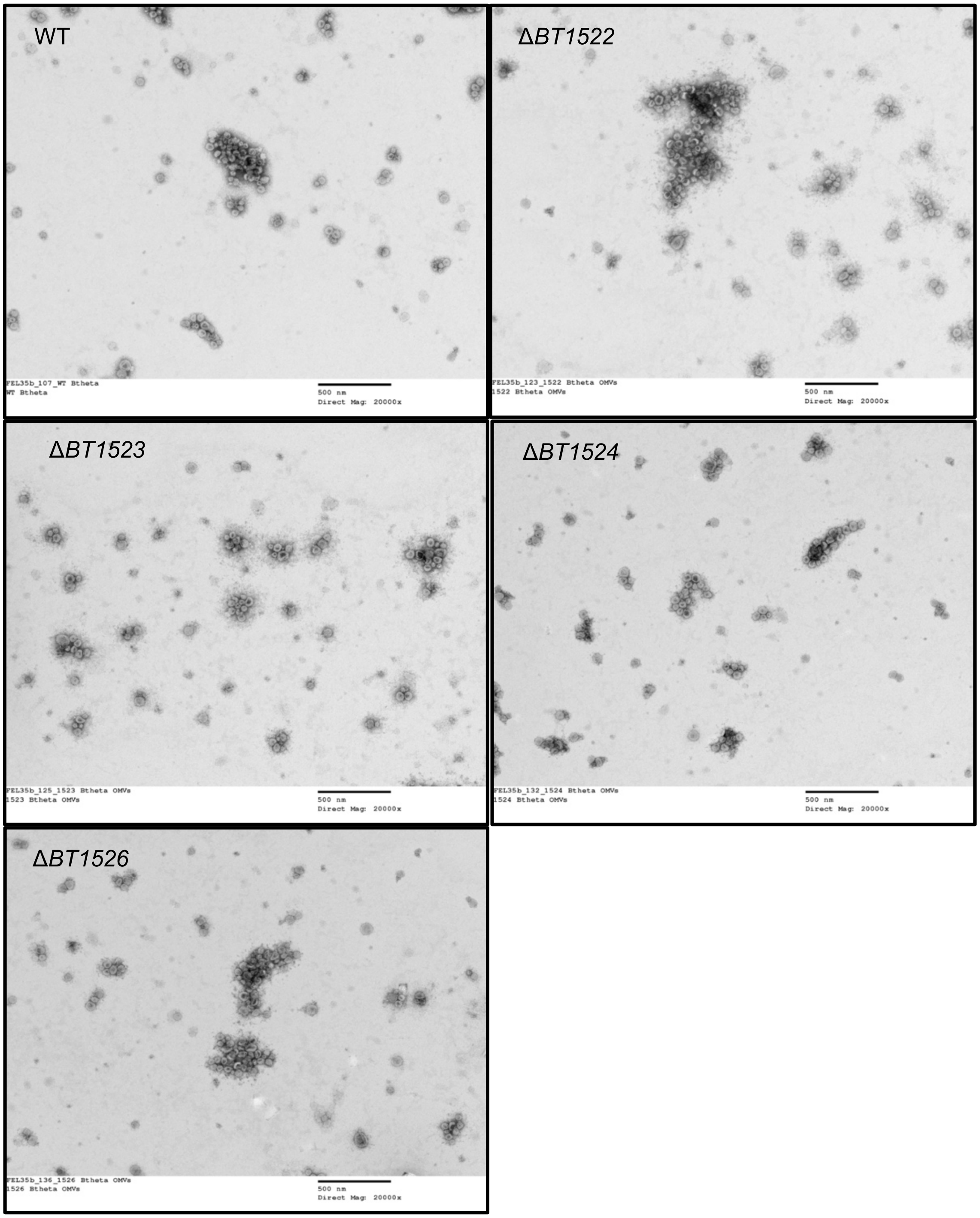

Figure S4. Lack of IPC and PI does not alter OMV secretion. OMV were prepared using early stationary phase liquid-grown cultures. OMV preparations were resuspended in PBS and imaged by TEM. Images were acquired by Wandy Beatty at the WUSTL Molecular Microbiology Imaging Facility. 20000x magnification is shown.

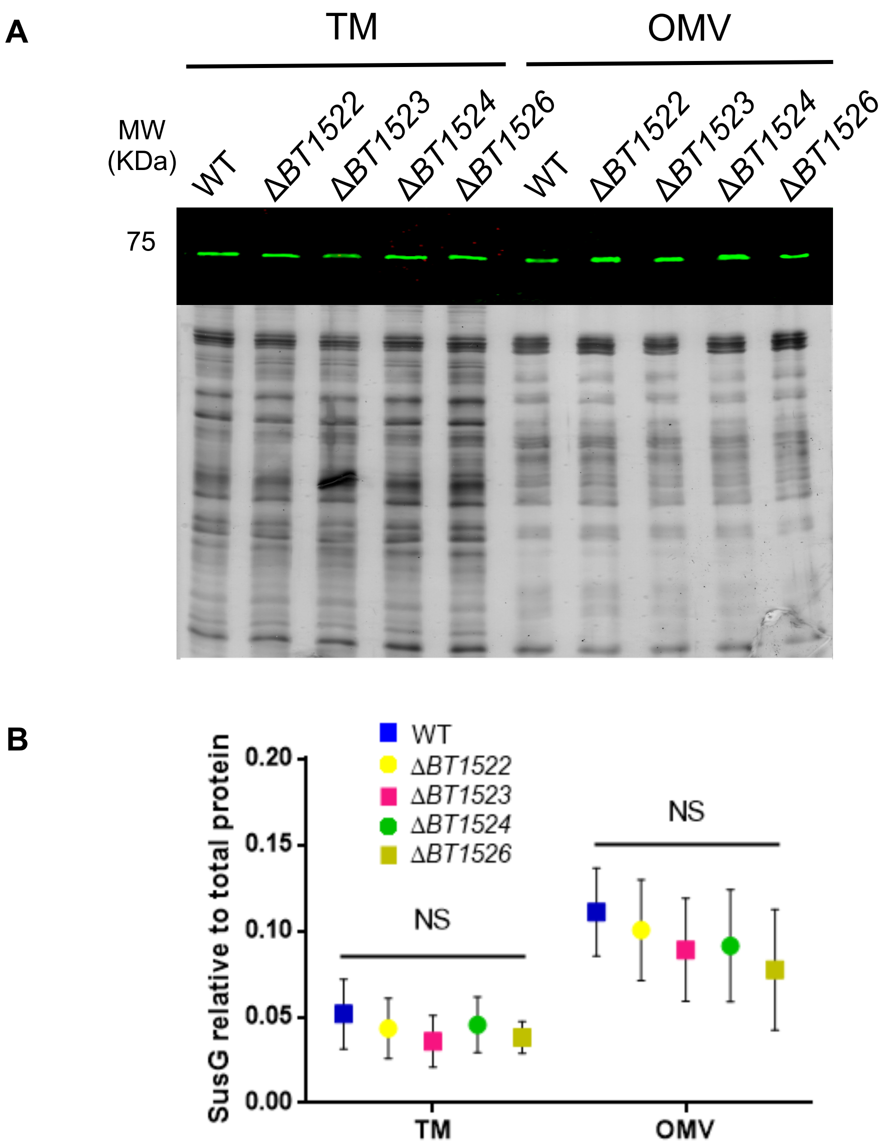

Figure S5. Lack of IPC does not affect cargo selection of OMV targeted proteins. TM and OMV fractions were prepared using early stationary phase liquid-grown cultures. TM and OMV preparations were resuspended in PBS normalizing by optical density (600 nm) for each strain. **A**. Western blot using anti-SusG antisera. **B.** SDS-PAGE revealed by silver stain as loading control. Graph shows means and error bars by SD of 3 biological replicates for each strain. Statistical significance was determined by *t*-test (p-value < 0.05).

Table S1. Lipid species identified in TM and OMV from *B. thetaiotaomicron* wild-type and Δ*BT1522-1526* strains.

Table S2. Strains, plasmids and oligonucleotides used for this study.

**Bacterial strains**			
**Species**	**Strain**	**Description**	**Reference/Source**
Escherichia coli	S17-1 λ*pir*	Conjugation donor strain for introduction of plasmids into *B. thetaiotaomicron*.	Gift from Goodman Lab
Bacteroides thetaiotaomicron	VPI-5482 Δ*tdk*	*tdk* deletion mutant, fluorodeoxyuridine (FudR) resistant. Wild-type strain for this study.	Koropatkin *et al.*, 2008
B. thetaiotaomicron	VPI-5482 Δ*spt*	*spt* deletion mutant. Sphingolipid biosynthesis deficient mutant.	This study
B. thetaiotaomicron	VPI-5482 Δ*bt1522*	*bt1522* deletion mutant.	This study
B. thetaiotaomicron	VPI-5482 Δ*bt1523*	*bt1523* deletion mutant.	This study
B. thetaiotaomicron	VPI-5482 Δ*bt1524*	*bt1524* deletion mutant.	This study
B. thetaiotaomicron	VPI-5482 Δ*bt1526*	*bt1526* deletion mutant.	This study
B. thetaiotaomicron	VPI-5482 Δ*bt1522* p1522c	*bt1522* deletion mutant, complemented.	This study
B. thetaiotaomicron	VPI-5482 Δ*bt1523* p1523c	*bt1523* deletion mutant, complemented.	This study
B. thetaiotaomicron	VPI-5482 Δ*bt1524* p1524c	*bt1524* deletion mutant, complemented.	This study
B. thetaiotaomicron	VPI-5482 Δ*bt1526* p1526c	*bt1526* deletion mutant, complemented.	This study
**Plasmids**			
**Name**	**Resistance**	**Description**	**Reference/Source**
pExchange-tdk	Amp/Erm	Suicide vector with FudR negative selection for gene deletion in *B. thetaiotaomicron* Δ*tdk*	Koropatkin *et al.*, 2008
pExchange-tdk/spt	Amp/Erm	Construct for deletion of *spt*	This study
pExchange-tdk/*bt1522*	Amp/Erm	Construct for deletion of *bt1522*	This study

pExchange-tdk/*bt1523*	Amp/Erm	Construct for deletion of *bt1523*	This study
pExchange-tdk/*bt1524*	Amp/Erm	Construct for deletion of *bt1524*	This study
pExchange-tdk/*bt1525*	Amp/Erm	Construct for deletion of *bt1525*	This study
pExchange-tdk/*bt1526*	Amp/Erm	Construct for deletion of *bt1526*	This study
pWW3867	Amp/Erm	Integrative plasmid harboring BT1311 promoter region, RBS phage, GFP, tag,Term, NBU2	Whitaker *et al.*, 2017
pWW3867-*bt1522*c	Amp/Erm	Construct for *B. thetaiotaomicron* Δ*tdk Δ1522* complementation	This study
pWW3867-*bt1523*c	Amp/Erm	Construct for *B. thetaiotaomicron* Δ*tdk Δ1523* complementation	This study
pWW3867-*bt1524*c	Amp/Erm	Construct for *B. thetaiotaomicron* Δ*tdk Δ1524* complementation	This study
pWW3867-*bt1526*c	Amp/Erm	Construct for *B. thetaiotaomicron* Δ*tdk Δ1526* complementation	This study

Oligonucleotides for cloning Up/Down fragments into pExchange-tdk.

* Caps letters indicate restriction enzyme cut sites, while underlined letters indicate overlapping sequences between Up/Down PCR fragments used for sewing.

Name	PCR product	Sequence
spt_UF	spt_Up	atatGTCGACttcattcttaaattcgctgaatacggac
spt_UR	spt_Up	gaattataatttattatactcatactcagatcattcgctg
spt_DF	spt_Down	tctgagtatgagtataataaattataattcgcagcatctctgaaaagctataaaaaagc
spt_DR	spt_Down	catatTCTAGAaaagactcattggaaatgtagtaggcaag
bt1522_UF	bt1522_Up	aaaGGATCCctatggaggaattatcaattgcattatc
bt1522_UR	bt1522_Up	ttgaattgtttttatcattaaggatgttattttaattag
bt1522_DF	bt1522_Down	aataacatccttaatgataaaaacaattcaatgaagaacaaataccgcaatatattcc
bt1522_DR	bt1522_Down	atatGTCGACcctaaggcatagcgaacgtctgatc
bt1523_UF	bt1523_Up	aaaGGATCCcagcattagacaacttttaactttcaac
bt1523_UR	bt1523_Up	atacctaaatctgtttttatctaaatcttc
bt1523_DF	bt1523_Down	gatttagataaaaacagatttaggtatttaaaataacatccttaatgataaaaac
bt1523_DR	bt1523_Down	atatGTCGACgtagtattaatcagataaacgaacagcc
bt1524_UF	bt1524_Up	aaaGGATCCgaagttagtaagctgtctgtaatcg
bt1524_UR	bt1524_Up	ccgtgcaactcttttcagccaatcacc
bt1524_DF	bt1524_Down	gatgggtgattggctgaaaagagttgcacggaagaaaacagaagatttagataaaaacag
bt1524_DR	bt1524_Down	atatGTCGACgtactcacaaggagtaaccagcac
bt1526_UF	bt1526_Up	aaaGGATCCcatccattcactgaaagtacgttatg
bt1526_UR	bt1526_Up	ttctattctattaaaattaaactaaaattgatg
bt1526_DF	bt1526_Down	gcatcaattttagtttaattttaatagaatagaattaaataagtggagagttgaaagttg

bt1526_DR	bt1526_Down	atatGTCGACgcatattataattccagaacaacgc
cbt1522F	1522compl	actccaaatctgtttttaaagaatgataaaaacaattcaaatgccgtcg
cbt1522R	1522compl	ttctcgagctaatcagctaggccaggaatatattgcggtatttgttcttcac
cbt1523F	1523compl	actccaaatctgtttttaaagaatgaattacagagattacttacagcaactg
cbt1523R	1523compl	ttctcgagctaatcagctaggaattagtcattcttcttttcgttcgctgt
cbt1524F	1524compl	actccaaatctgtttttaaagaatgggtgattggctgaaaagagttg
cbt1524R	1524compl	ttctcgagctaatcagctaggcttctgttttctttcaatttcttttaaacaagcttc
cbt1526F	1526compl	actccaaatctgtttttaaagaatgaaacaagaaattaaacccgctactgga
cbt1526R	1526compl	ttctcgagctaatcagctaggctttcaactctcaactttcaactctccac
pWW3867L1	pWW3867lin	tctttaaaaacagatttggagtgcaaagttacg
pWW3867L2	pWW3867lin	cctagctgattagctcgagaaggc

Koropatkin, N. M., Martens, E. C., Gordon, J. I., & Smith, T. J. (2008). Starch catabolism by a prominent human gut symbiont is directed by the recognition of amylose helices. *Structure (London, England : 1993)*, *16*(7), 1105–1115. https://doi.org/10.1016/j.str.2008.03.017

Whitaker, W. R., Shepherd, E. S., & Sonnenburg, J. L. (2017). Tunable Expression Tools Enable Single-Cell Strain Distinction in the Gut Microbiome. *Cell*, *169*(3), 538–546.e12. https://doi.org/10.1016/j.cell.2017.03.041
